# Intestinal FXYD12 and sodium-potassium ATPase: A comparative study on two euryhaline medakas in response to salinity changes

**DOI:** 10.1371/journal.pone.0201252

**Published:** 2018-07-27

**Authors:** Wen-Kai Yang, An-Di Hsu, Chao-Kai Kang, Ivan Pochou Lai, Pei-Shao Liao, Tsung-Han Lee

**Affiliations:** 1 Department of Life Sciences, National Chung Hsing University, Taichung, Taiwan; 2 The iEGG and Animal Biotechnology Center, National Chung Hsing University, Taichung, Taiwan; 3 Bachelor Degree Program in Animal Healthcare, Hungkuang University, Taichung, Taiwan; 4 Tainan Hydraulics Laboratory, National Cheng Kung University, Tainan, Taiwan; 5 National Taichung First Senior High School, Taichung, Taiwan; Institut National de la Recherche Agronomique, FRANCE

## Abstract

FXYD proteins are the regulators of sodium-potassium ATPase (Na^+^/K^+^-ATPase, NKA). In teleosts, NKA is a primary driving force for the operation of many ion transport systems in the osmoregulatory organs (e.g. intestines). Hence, the purpose of this study was to determine the expression of FXYD proteins and NKA α-subunit in the intestines of two closely related medakas (*Oryzias dancena* and *O*. *latipes*), which came from different salinity habitats and have diverse osmoregulatory capabilities, to illustrate the association between NKA and FXYD proteins of two medaka species in response to salinity changes. The results showed that the *fxyd12* mRNA was the most predominant in the intestines of both medakas. The association of FXYD12 and NKA in the intestines of the two medaka species was demonstrated via double immunofluorescent staining and co-immunoprecipitation. Upon salinity challenge, the localization of FXYD12 and NKA was similar in the intestines of the two medaka species. However, the expression profiles of intestinal FXYD12 and NKA (mRNA and protein levels), as well as NKA activity differed between the medakas. These results showed that FXYD12 may play a role in modulating NKA activity in the intestines of the two medakas following salinity changes in the maintenance of internal homeostasis. These findings contributed to knowledge of the expression and potential role of vertebrate FXYD12, the regulators of NKA, upon salinity challenge.

## Introduction

Salinity adaptation of euryhaline teleosts that inhabit fresh water (FW) or seawater (SW) is a complex process, involving a set of physiological responses by osmoregulatory organs (i.e. gills, kidneys, and intestines) for ionoregulation in response to environmental challenges [1, 2]. Euryhaline teleosts have developed excellent osmoregulatory mechanisms in a wide variety of environmental salinities, and their adaptive responses to these variations has resulted in different capabilities [1, 3]. In FW (i.e. the hypoosmotic environment), teleosts decrease raised moisture and diluted ion content by absorbing external ions through the gills and gut and diluting their urine in the kidney. Conversely, teleosts living in SW (i.e. the hyperosmotic environment) maintain their homeostasis by drinking seawater, absorbing salts and water through the intestines, and secreting ions actively through the gills [1, 4].

In the osmoregulatory processes, sodium-potassium ATPase (Na^+^/K^+^-ATPase, NKA) is an active pump providing the primary driving force for triggering many ion transport systems in the osmoregulatory organs of teleosts [3, 4]. NKA is a P-type ATPase consisting of an (αβ)_2_ protein complex. The molecular weights of the catalytic α-subunit and the smaller glycosylated β-subunit are approximately 100 and 55 kDa, respectively [5]. Many euryhaline teleosts exhibit acclimated changes in their NKA responses following salinity variation [1, 3]. Therefore, the NKA expression could be a useful indicator of ion transport activity in the osmoregulatory organs of teleosts [6, 7]. In general, increased NKA expression indicates a greater driving force has been provided in response to an increased demand for ion uptake or secretion [2, 8, 9]. NKA expression and/or activity should therefore be modulated by a variety of mechanisms under changing physiological conditions [10, 11].

Among NKA regulators of euryhaline teleosts, the FXYD proteins were found to be novel regulators [12, 13]. Compared to teleosts, the FXYD protein family has been more widely studied in mammals and elasmobranchs. Most FXYD proteins are known to exhibit tissue-specific distribution and affect kinetic properties of NKA by interacting with NKA in specific ways [14, 15]. Recently, in teleosts, FXYD proteins were reported in several species, and multiple FXYD proteins have been identified [16–23]. Moreover, in the osmoregulatory organs, salinity-dependent profiles (mRNA and/or protein) of certain FXYD members, especially gill FXYD11 and renal FXYD12, suggested that they have physiological significance in osmoregulation and play crucial roles in modulating the NKA activity/expression [16–25].

The two organisms selected for this study were the Indian medaka and Japanese medaka (*Oryzias dancena* and *O*. *latipes*, respectively) because of their closely phylogenetic relationship and diverse osmoregulatory capabilities [26, 27]. The Indian medaka and Japanese medaka, residing in brackish water (BW) and FW environments, respectively, are both euryhaline teleosts [27, 28]. The Indian medaka exhibited better tolerance than the Japanese medaka in SW, including survival rates of adult fish and hatching rates of fertilised eggs [26, 27]. Moreover, the profiles of both branchial and renal NKA activity were different between the two medakas following salinity challenges [25, 29]. Regarding FXYD proteins, similar tissue-specific expression was found between the two medakas [23]. However, in gills and kidneys, the salinity-dependent expression profiles of FXYD proteins (mRNA and/or protein levels) differed between the two medakas [23, 25]. Therefore, these two closely-related euryhaline medakas from different natural habitats are good models for comparative studies on their osmoregulatory mechanisms including FXYD and NKA.

In the two medakas, the expression and potential role of FXYD proteins have been investigated in gills and kidneys [23, 25], but not in intestines. The intestine possesses a large surface area with which to perform both secretory and absorptive functions via villi and microvilli and it plays a key role in absorption of water to avoid dehydration, particularly in SW teleosts [2, 30, 31]. Our previous study has identified seven members of the FXYD protein family (i.e. FXYD5-9, FXYD11, and FXYD12) in each medaka species [23]. Among them, FXYD12 was predominant in the kidney and intestine of both medakas ([23, 25], S1 Fig), and thus it was speculated to play a role in the modulating NKA activity/expression in both osmoregulatory organs of the two medakas upon salinity challenge. The goals of this study were to (i) compare the profiles of intestinal FXYD12 between these two medakas via localization and expression of FXYD12 and NKA, as well as their interaction; and (ii) clarify the potential roles of FXYD proteins in modulating NKA activity in the osmoregulatory organs (i.e. gill, kidney, and intestine) of the two medakas upon salinity challenge.

## Materials and methods

### Fish and experimental environments

Adult Indian medaka and Japanese medaka (HI strain) were both bred in the laboratory and both with the standard length of 2–3 cm. For the experiments, the two medakas were acclimated to either FW, BW (15‰), or SW (35‰) for at least one month. In all groups, water temperature and photoperiod were held at 28 ± 1°C and under a 14-h/10-h light/dark cycle, respectively [23, 25]. BW and SW were prepared using local fresh tap water (i.e. aerated dechlorinated FW) with added Instant Ocean synthetic sea salt (Aquarium Systems, Mentor, OH, USA). The fish were fed daily with a commercial pellet diets. In the following experiments, the fish were fasted for one day and were then anesthetised (100–200 mg L^-1^ MS-222; Sigma, St Louis, MO, USA) before sampling. The protocols used in this study were reviewed and approved by the Institutional Animal Care and Use Committee of the National Chung Hsing University (approval no. 96–48) and were carried out in accordance with the approved guidelines.

### Sample preparation

Following anaesthesia, whole intestines were dissected, put into microcentrifuge tubes, frozen with liquid nitrogen, and then stored immediately at -80°C until total RNA extraction for mRNA assay or homogenization for protein assay. For paraffin- and cryo-sectioning, the dissected intestines were immediately fixed in Bouin’s fluid (Sigma) and 10% neutral buffered formalin (Taiwan Burnett, Taipei, Taiwan), respectively.

### Total RNA extraction and reverse transcription

The methods used in this study were identical with those of our previous study [25]. The concentration and integrity of the extracted RNA were determined with a NanoDrop 2000 (Thermo, Wilmington, DE, USA) and verified by 0.8% agarose gel electrophoresis, respectively. Extracted RNA samples and cDNA products were stored at -80°C and -20°C, respectively, until use.

### Quantitative real-time polymerase chain reaction (Q-PCR)

Q-PCR was carried out with the MiniOpticon real-time PCR system (Bio-Rad, Hercules, CA, USA). The PCR assays contained 10 μL of 2× SYBR Green Supermix (Bio-Rad), 2 μL of either 1 μM target gene primers (*fxyd* or *nka* α-subunit) or internal control primers (*β-actin* or *ribosomal protein L7* for salinity-effect analyses or others, respectively), and 8 μL of cDNA (1000× dilution). Probe constructs for Q-PCR and their detailed information are shown in S1 Table, and these primers have been used successfully in these two medaka species [23, 25, 32]. The PCR analysis and the calculation formula were described in previous studies [22, 33].

### Antibodies

Primary antibodies were: (1) FXYD12: a rabbit polyclonal antibody (LTK BioLaboratories, Taoyuan, Taiwan) targeting the C-terminus of medaka FXYD12 (S2 Fig); (2) NKA: a mouse monoclonal antibody (α5; Developmental Studies Hybridoma Bank, Iowa City, IA, USA) against the α-subunit of avian NKA; and (3) actin: a rabbit polyclonal antibody (sc-1616-R; Santa Cruz Biotechnology, Santa Cruz, CA, USA) against the C-terminus of human actin, as a loading control for immunoblotting. The specificity and related information for these antibodies have been demonstrated and described previously [25, 29].

The secondary antibodies employed for immunofluorescent staining were Alexa-Fluor-546-conjugated goat anti-rabbit IgG and Alexa-Fluor-488-conjugated goat anti-mouse IgG (Molecular Probes, Eugene, OR, USA). For immunoblotting, the secondary antibodies were horseradish-peroxidase-conjugated goat anti-mouse IgG and anti-rabbit IgG (Pierce, Rockford, IL, USA).

### Paraffin section and immunohistochemical staining

The methods used herein were modified from Yang et al. [9, 25]. Briefly, the deparaffinised sections (5-μm thickness) were incubated with 3% hydrogen peroxide for 10 min to inactivate endogenous peroxidase. After washing with phosphate-buffered saline (PBS; 137 mM NaCl, 3 mM KCl, 10 mM Na_2_HPO_4_, 2 mM KH_2_PO_4_, pH 7.4), the sections were incubated with the primary antibody (α5 or FXYD12; 200× or 400× dilution in PBS, respectively) or negative control (PBS only) before analysis using a commercial kit (PicTure™; Zymed, South San Francisco, CA, USA). Afterward, the slides were counterstained with haematoxylin (Merck, Darmstadt, Germany). Finally, the immunosignals were imaged by an optical microscope (BX50; Olympus, Tokyo, Japan) equipped with the cooling charge-coupled device camera (DP72; Olympus) using CellSens standard v1.4 software (Olympus).

### Cryosection and double immunofluorescence staining

The procedures were modified from previous studies by our group [25, 34]. After fixation, tissues were permeated with methanol at -20°C overnight and then infiltrated with Tissue-Tek O.C.T. compound (Sakura, Torrance, CA, USA) overnight at 4°C. After cryosectioning, sections (5-μm) were kept at -20°C.

For double staining, cryosections were rinsed by PBS and then pre-incubated with PBS containing 5% bovine serum albumin (Bio Basic, Markham, Canada) for 30 min. Afterward, cryosections were incubated with FXYD12 antibody (400× dilution in PBS) at 4°C overnight. Next, sections were incubated with the secondary antibody (Alexa-flour-546 antibody) for 2 h. Subsequently, sections were incubated with NKA antibody (200× dilution in PBS) for 2 h followed by the secondary antibody (Alexa-flour-488 antibody) for 2 h. The immunoreactions were observed using a laser scanning confocal microscope (FV1000; Olympus) or a fluorescent microscope (BX50; Olympus) with DP72 camera (Olympus) as described in the previous paragraph.

### Preparation of intestinal homogenates

Sample scrapings were soaked with homogenization medium (SEID buffer) [25] and proteinase inhibitor (#11836145001; Roche, Indianapolis, IN, USA), and then homogenized with a Polytron PT1200E (Kinematica, Lucerne, Switzerland) at maximum speed of 30 s on ice. After centrifuging at 5000 ×*g* and 4°C for 5 min, the supernatants were collected and then used immediately for protein concentration measurements or determination of NKA activity or then stored at -80°C for immunoprecipitation analysis or immunoblotting. Protein concentrations were measured using the BCA Protein Assay (#23225; Pierce) following the manufacturer’s instructions.

### Specific activity of NKA

NKA activity was determined using the NADH-linked method [35] with modifications for medakas [25, 29]. The methods used herein were identical with our previous study [25]. The NKA activity was calculated as the difference in slope of ATP hydrolysis (i.e. NADH reduction) with or without ouabain (0.75 mM; Sigma; a specific NKA inhibitor), and was expressed as μmol ADP per mg protein per h.

### Immunoblotting

Immunoblotting procedures were conducted as described previously [25, 36]. In brief, sample homogenates (10 μg of protein/lane) prepared as stated previously were mixed with denaturing buffer and then heated at 60°C for 15 min. After electrophoresis and transfer of the proteins from the gels to polyvinylidene fluoride membranes (Millipore, Bedford, MA, USA), the blots were pre-incubated for 2 h in 5% nonfat dried milk in PBST (PBS with 0.05% Tween 20) to minimize non-specific binding. Subsequently, the membranes were incubated overnight at 4°C with FXYD12 antibody (60000× dilution), NKA antibody (2500× dilution), or actin antibody (6000× dilution) in PBST containing 1% bovine serum albumin and 0.05% sodium azide (Sigma), followed by incubation with the appropriate secondary antibody. Next, the blots were developed with the Immobilon Western Chemiluminescent HRP substrate (Millipore) and then imaged using a cooling charge-coupled device camera (ChemiDoc XRS+; Bio-Rad) with Quantity One v4.6.8 software (Bio-Rad). Immunosignals were finally converted to numerical values using Image Lab v3.0 (Bio-Rad) for quantifying and comparing the relative protein amounts of intestinal samples among all groups.

### Co-immunoprecipitation

Total intestinal lysates were used. Immunoprecipitation with either the anti-FXYD12, pre-serum (as negative control for FXYD12), or anti-NKA was performed with the ImmunoCruz^TM^ IP/WB Optima System (sc-45042 and sc-45043; Santa Cruz Biotechnology) following the manufacturer’s manual [25, 32]. These immunoprecipitation solutions were then analyzed using immunoblotting as described above for confirming the protein interaction between intestinal FXYD12 and NKA.

### Statistical analysis

The values were shown as means ± standard error. The Shapiro-Wilk’s test and Levene's test were applied to test normality and homogeneity of variance, respectively, for all data before further analysis. One-way analysis of variance and post-hoc Tukey’s test were carried out, and statistical significant was considered at *P* < 0.05. All statistical analysis was performed with SPSS 20.0 (SPSS, Chicago, IL, USA).

## Results

### Levels of detected *fxyd* genes in the intestines of two medakas

**Fig 1:**
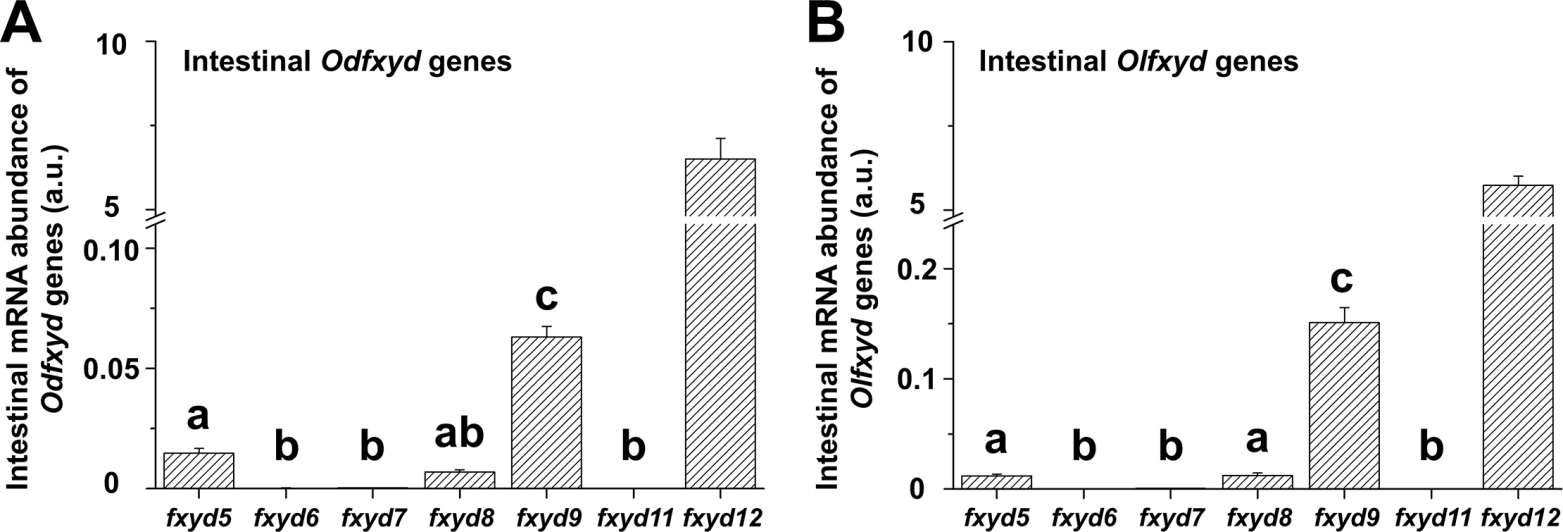
**Levels of intestinal *fxyd* mRNA in the Indian medaka (Od; A) and the Japanese medaka (Ol; B).** The values are means ± SEM (total N = 12; N = 4 in the freshwater, brackish water, and seawater group, respectively). Different letters indicate significant differences among *fxyd* genes, excluding *fxyd12* (*P* < 0.05). A.u., arbitrary units.

Similar profiles of the intestinal *fxyd* mRNA were found between the Indian medaka and Japanese medaka (*Odfxyd* and *Olfxyd*, respectively) (Fig 1). In the intestine of both medakas, among these *fxyd* genes, the *fxyd12* and *fxyd9* were the most predominant and the second, respectively. The intestinal mRNA levels of *Odfxyd12* and *Olfxyd12* were approximately 108- and 40-fold higher than those of *Odfxyd9* and *Olfxyd9*, respectively. Meanwhile, the low abundance of intestinal *fxyd6*, *fxyd7*, and *fxyd11* mRNA was observed in both medakas (Fig 1).

### Distribution of FXYD12 and NKA in the intestines of two medakas

**Fig 2:**
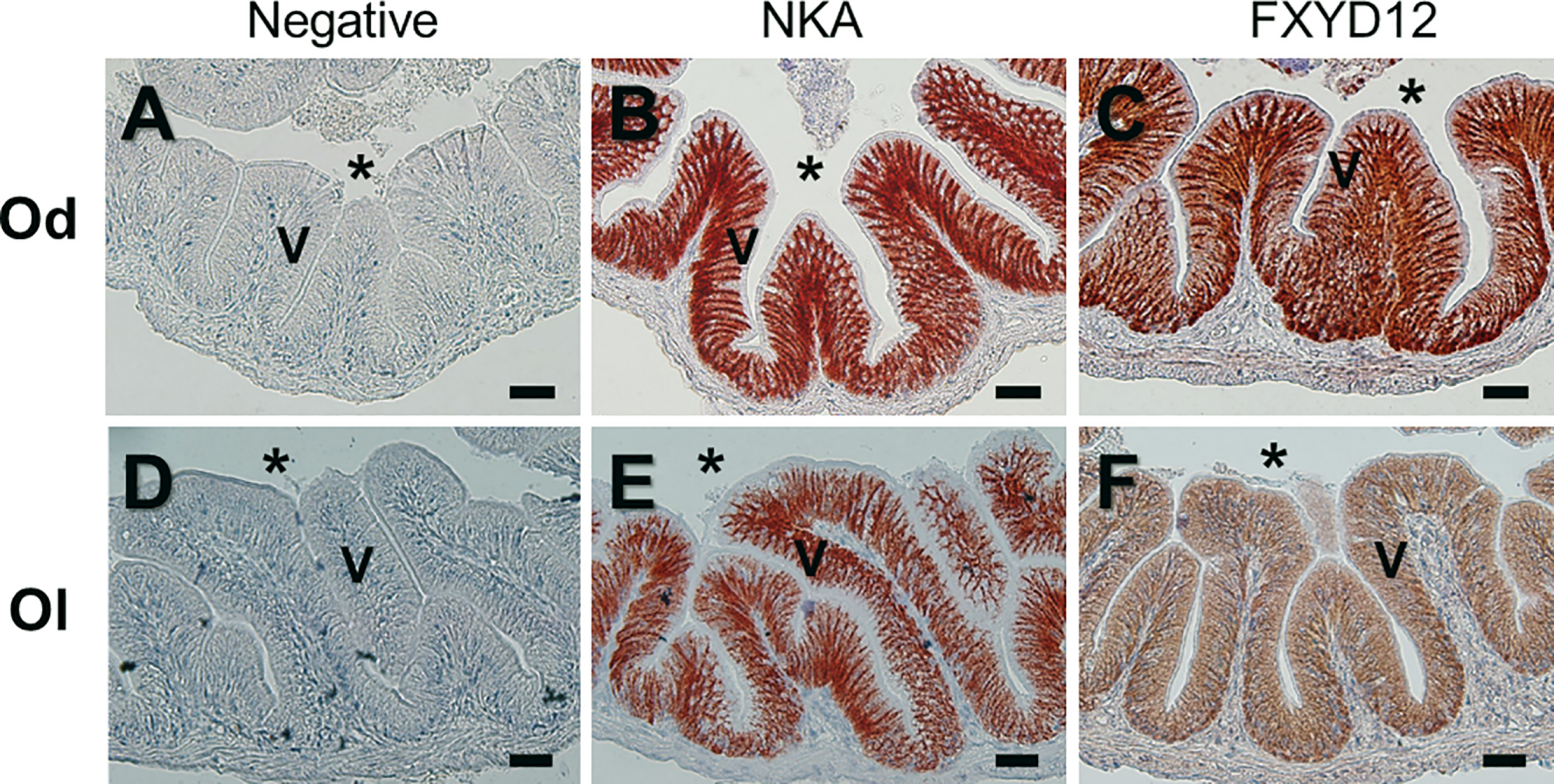
**Immunohistochemical localization of NKA α-subunit (NKA) and FXYD12 in paraffin cross sections of intestines of the brackish water-acclimated Indian medaka (Od; A-C) and fresh water-acclimated Japanese medaka (Ol; D-E).** Immunosignals of NKA (**B**, **E**) and FXYD12 (**C**, **F**) were both detected in the basolateral membrane of intestinal epithelium, compared with the negative control (**A**, **D**). V, villus; *, lumen. Scale bar: 20 μm.

**Fig 3:**
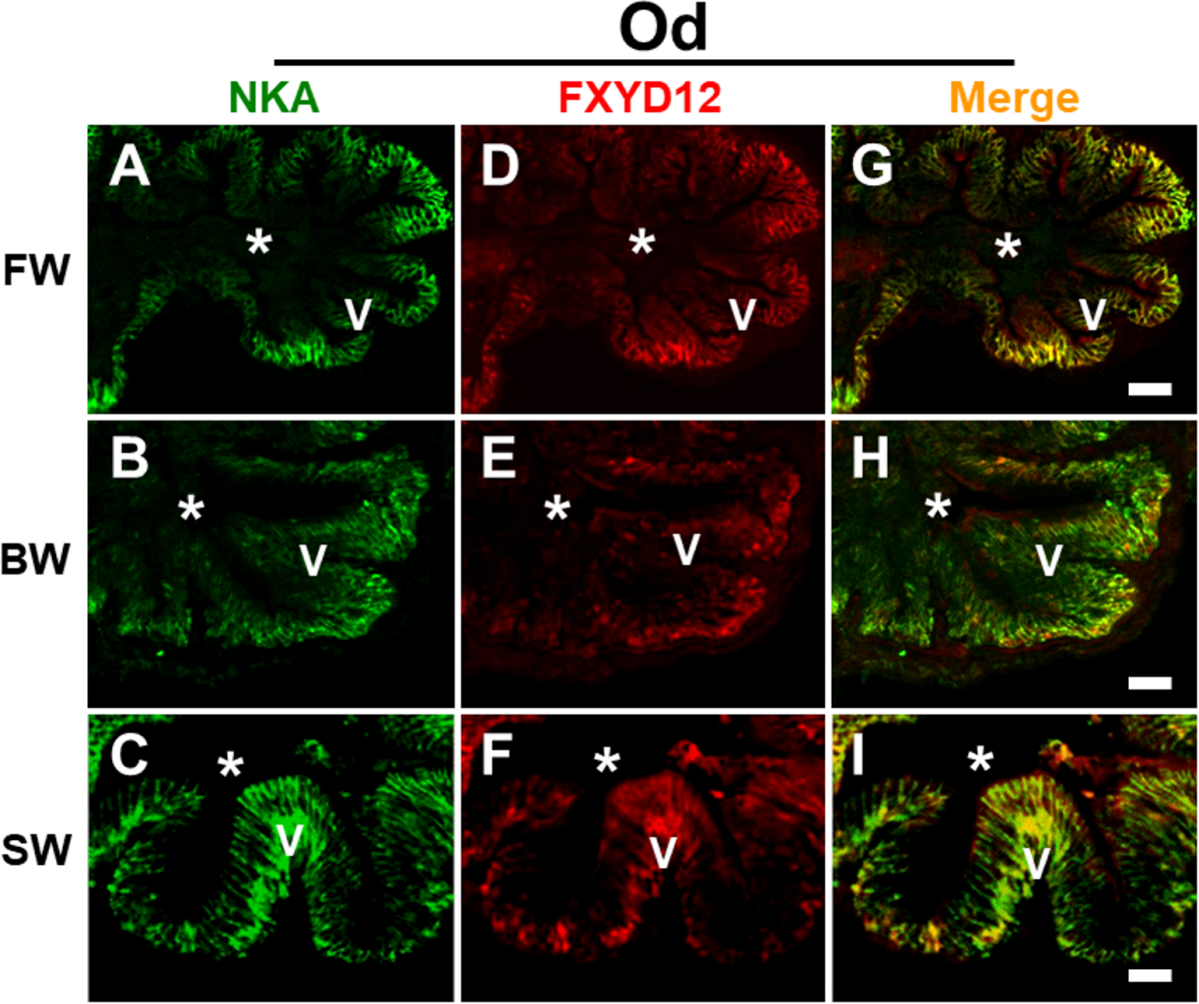
**Double immunofluorescence staining of NKA α-subunit (NKA; green; A-C) and FXYD12 (red; D-F) in intestinal cross-cryosections of the Indian medaka (Od).** The merged images (yellow; **G**, **H**, **I**) revealed that FXYD12 colocalised in the basolateral membrane of NKA-immunoreactive cells in the fresh water- (FW; **A**, **D**, **G**), brackish water- (BW; **B**, **E**, **H**), and seawater- (SW; **C**, **F**, **I**) acclimated fish. V, villus; *, lumen. Scale bar: 20 μm.

**Fig 4:**
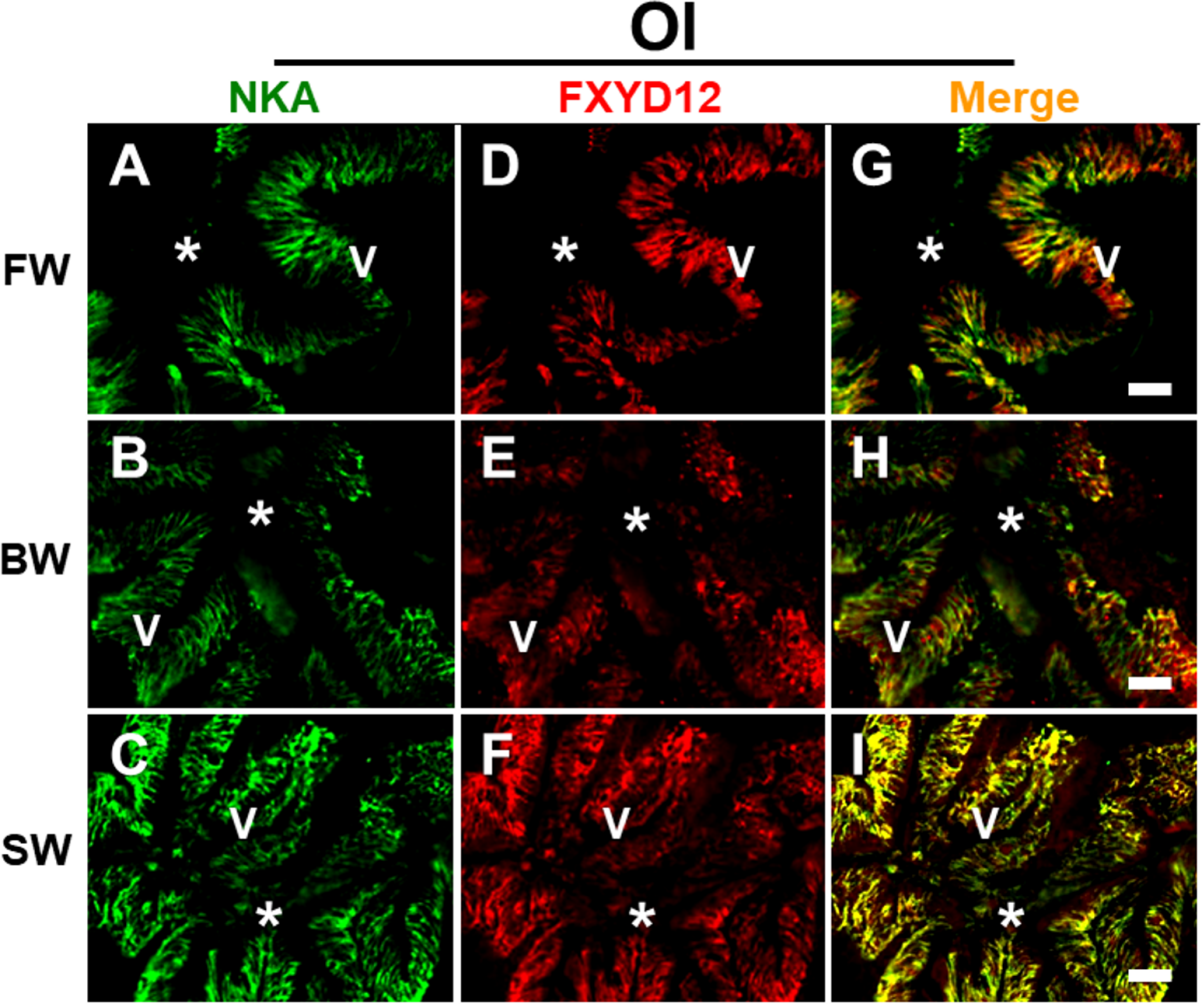
**Double immunofluorescence staining of NKA α-subunit (NKA; green; A-C) and FXYD12 (red; D-F) in intestinal cross-cryosections of the Japanese medaka (Ol).** The merged images (yellow; **G**, **H**, **I**) revealed that FXYD12 colocalised to the basolateral membrane of NKA-immunoreactive cells in the fresh water- (FW; **A**, **D**, **G**), brackish water- (BW; **B**, **E**, **H**), and seawater- (SW; **C**, **F**, **I**) acclimated fish. V, villus; *, lumen. Scale bar: 20 μm.

**Fig 5:**
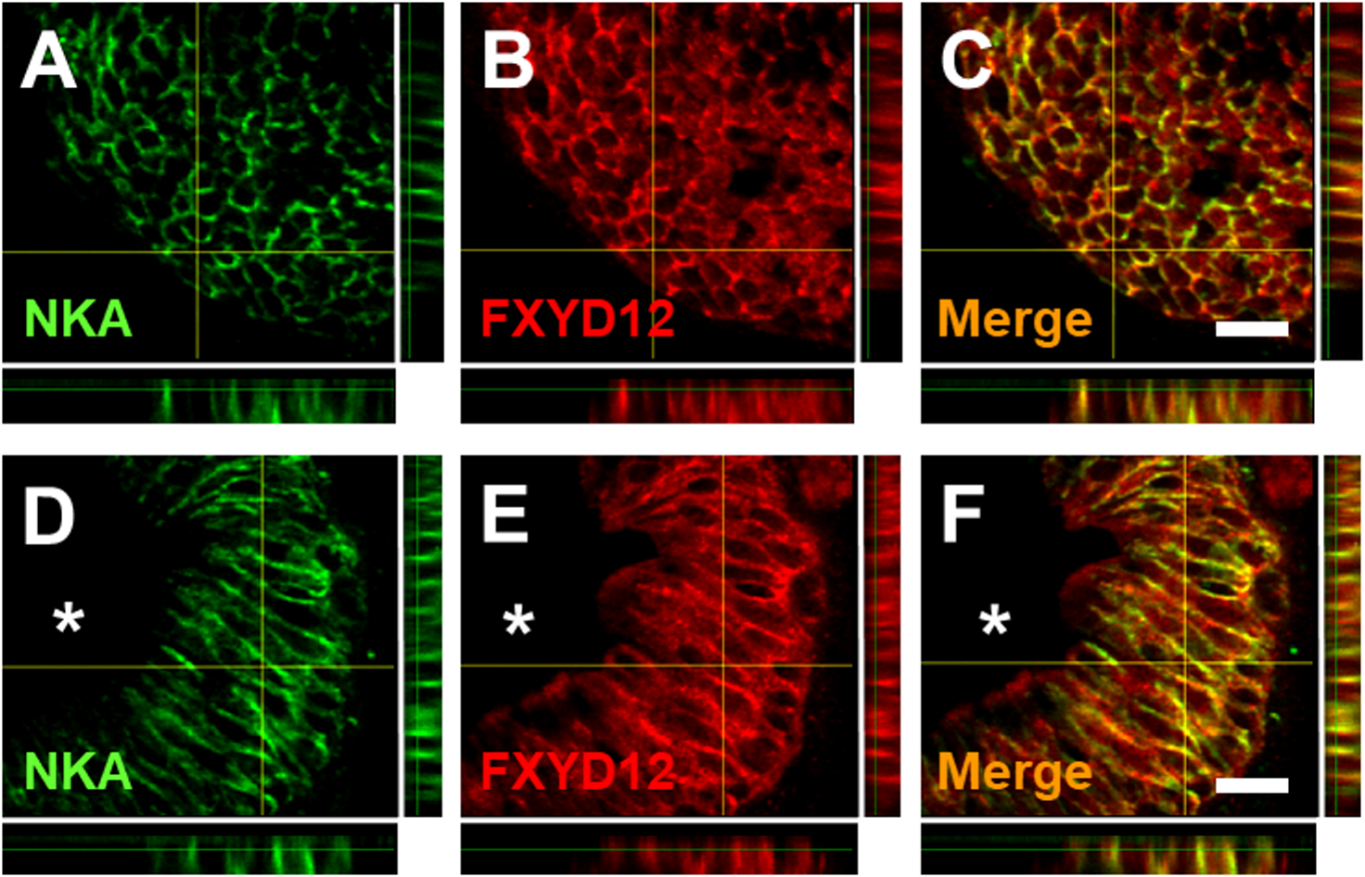
**Confocal 3D micrographs of double immunofluorescence staining of NKA α-subunit (NKA; green; A, D) and FXYD12 (red; B, E) in the cryosection of the brackish water-acclimated Indian medaka intestines.** Immunosignals of NKA and FXYD12 were both detected in epithelial cell of intestinal villi. The results were similar between the cross section (**A-C**) and longitudinal section (**D-F**) of the epithelial cells. The merged image (yellow; **C**, **F**) revealed that FXYD12 colocalised to the basolateral membrane of NKA-immunoreactive cells. *, lumen. Scale bar: 10 μm.

FXYD12 and NKA were immunohistochemically stained in the intestines of the two medakas (Fig 2). Negative control showed non-specific staining or overstaining as a comparison (Fig 2A and 2D). Based on the results of intestinal histology (S3 Fig), in the Indian medaka, both NKA and FXYD12 were observed in the basolateral membrane of intestinal epithelium (Fig 2B and 2C). Meanwhile, in the Japanese medaka, the results showed that NKA was also found in the basolateral membrane of intestinal epithelium (Fig 2E; S3 Fig). Although the FXYD12 signals were slightly weak, the FXYD12 also expressed predominately in the basolateral membrane of intestinal epithelium in the Japanese medaka (Fig 2F). On the other hand, in double immunofluorescence staining, the results further revealed that most FXYD12 was localised in the basolateral membrane of NKA-immunoreactive (-IR) cells (Figs 3 and 4). The results were similar among all salinity groups. Confocal 3D micrographs of cross section and longitudinal section of the microvilli in intestines of the BW-acclimated Indian medaka showed clear images (yellow) to confirm the co-localization of FXYD12 and NKA (Fig 5). These results demonstrated that FXYD12 was localized in the basolateral membranes of NKA-IR cells in the intestinal epithelium of both two medakas.

### Association between intestinal FXYD12 and NKA in the two medakas

**Fig 6:**
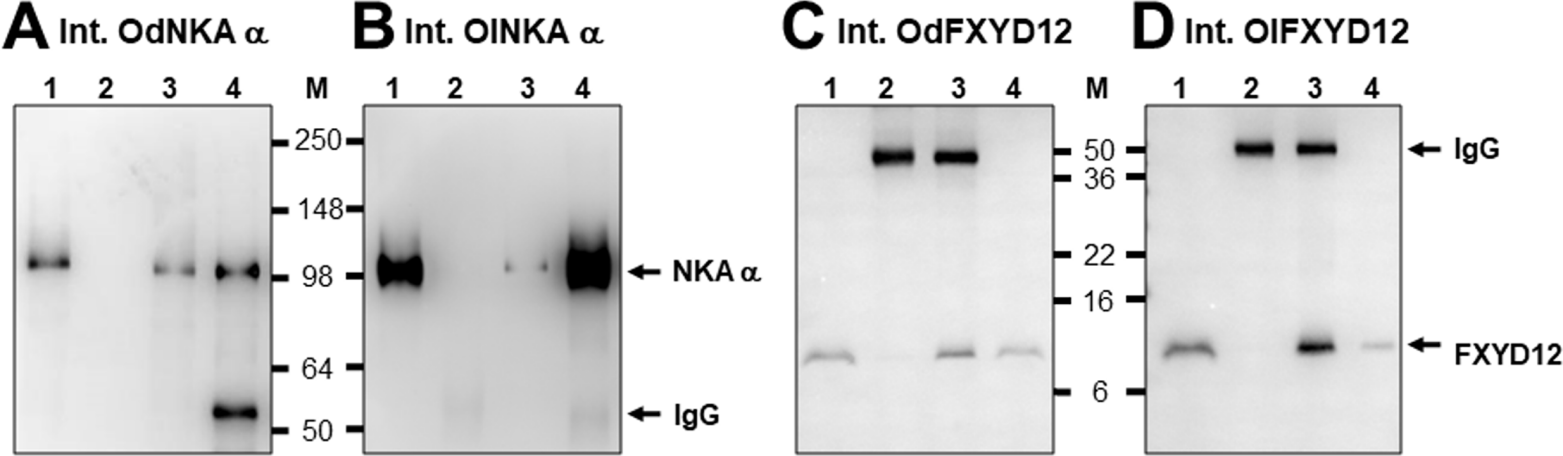
**Co-immunoprecipitation of FXYD12 and NKA α-subunit (NKA α) in the intestines (Int.) of the brackish water-acclimated Indian medaka (Od; A, C) and the fresh water-acclimated Japanese medaka (Ol; B, D).** Immunoreactive bands of NKA α (**A**, **B**) or FXYD12 (**C**, **D**) were detected at 100 or 10 kDa, respectively. Lane 1, positive control (total intestinal lysates); lane 2, negative immunoblot control using pre-immune serum for immunoprecipitation; lanes 3 and 4, experimental group using antibodies (FXYD12 and NKA α, respectively) for immunoprecipitation. The 55 kDa bands in lanes 2–4 are the IgG heavy chains of the FXYD12 or NKA α antibody. M, marker (kDa).

The interaction between the two proteins in the intestines of the two medakas was examined by co-immunoprecipitation (Fig 6). The results revealed that when NKA and FXYD12 were precipitated, and the immunobands were found at 10 and 100 kDa corresponding to the molecular masses of the FXYD12 and NKA α-subunit, respectively. Meanwhile, the efficiency of immunoprecipitation was demonstrated via the positive and negative controls (lanes 1 and 2 of Fig 6, respectively). The results of protein interaction were similar between the two medaka species (Fig 6).

### Salinity effects on the expression profiles of intestinal FXYD12 and NKA

**Fig 7:**
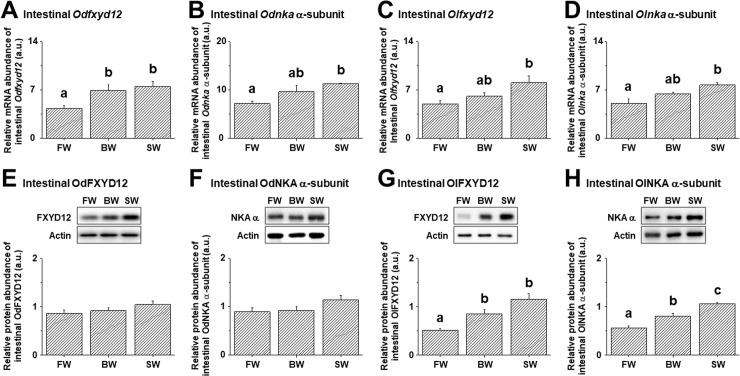
**Effects of salinity on mRNA levels (A-D) and protein abundance (E-H) of intestinal FXYD12 and NKA α-subunit (NKA α) in the Indian medaka (Od) and the Japanese medaka (Ol).** The values are means ± SEM (N = 6 or 10 in **A-D**,**H** or **E-G**, respectively). Dissimilar letters indicate significant differences among various salinity groups (*P < 0*.*05*). Actin was used as an internal control for the immunoblots. FW, fresh water; BW, brackish water; SW, seawater.

**Fig 8:**
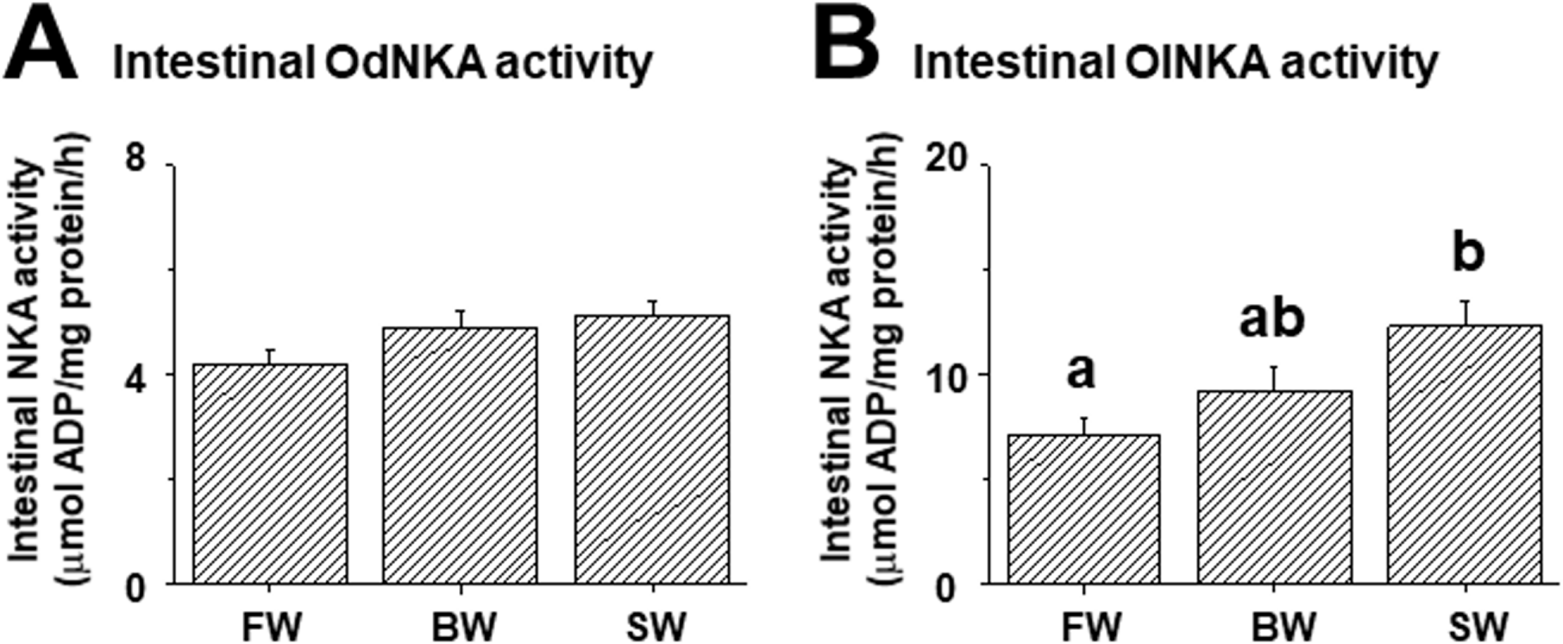
**Effects of salinity on intestinal NKA activity in the Indian medaka (Od; A) and the Japanese medaka (Ol; B).** The values are means ± SEM (N = 6). Dissimilar letters indicate significant differences among various salinity groups (*P < 0*.*05*). FW, fresh water; BW, brackish water; SW, seawater.

In mRNA levels, the results of both medaka intestines showed similar patterns of FXYD12 and NKA that increased with environmental salinities (Fig 7A–7D). Meanwhile, in protein levels, as well as NKA activities, the expression profiles of both FXYD12 and NKA differed between medakas (Figs 7E–7H and 8). In the intestines of Indian medaka, the expression profiles (i.e. protein and activity) of both FXYD12 and NKA were not significantly different upon salinity challenge (Figs 7E, 7F and 8A). On the other hand, the three profiles (i.e. expression of both two proteins and NKA activity) increased with environmental salinities in the intestines of the Japanese medaka (Figs 7G, 7H and 8B).

## Discussion

The intestines of teleosts are crucial for the absorption of nutrients, ions, and water. They play a critical role in osmoregulation via water reabsorption to compensate for osmotic water loss in the hyperosmotic environment [2, 30]. Although the mechanisms are not clear, water reabsorption can be performed through ion transport proteins, tight junction, water channels, and/or bicarbonate cycle [31, 37]. In these processes, NKA is crucial for maintaining intracellular homeostasis via its activity to increase transport of ions and water by numerous transporters and channels [30, 37].

In this study, different expression patterns of intestinal NKA activity were observed between the two medakas. In the Japanese medaka, intestinal NKA activity increased with environmental salinity. Similar results were found in the other teleosts [16, 38–40]. Meanwhile, in certain teleosts including the California Mozambique tilapia (*Oreochromis mossambicus* x *O*. *urolepis hornorum*) [41], black porgy (*Acanthopagrus schlegeli*) [42], green sturgeon (*Acipenser medirostris*) [43], and Indian medaka (this study), no significant difference of intestinal NKA activity was found between FW and SW groups. Among these species, in the intestines of the California Mozambique tilapia and black porgy, the NKA activity increased significantly with hypersaline SW (75 and 45‰, respectively) [41, 42]. In telesots, the osmoregulatory capabilities may be related to their evolutionary histories, life histories, and/or natural habitats [1, 3, 27]. Previous studies reported that the Indian medaka have better osmoregulatory capability compared to that of the Japanese medaka in SW [26, 27]. Hence, the expression profiles of intestinal NKA activity may vary in teleostean species with different osmoregulatory capabilities.

Several modulatory mechanisms of NKA activity have been reported, including NKA expression, NKA isoform compositions, and FXYD proteins [10, 44]. Among them, FXYD proteins modulate NKA expression via interacting with NKA [10, 11, 15]. FXYD proteins of teleosts in response to salinity challenge were recently identified and investigated. Previous studies indicated that the FXYD12 was expressed principally in kidneys and/or intestines of teleosts including the Indian medaka, Japanese medaka [23, 25] and others [16, 18, 20]. In mammals, higher expression of FXYD3 and FXYD4 were reported in the gastrointestinal tract, such as the intestine, colon, and rectum [10, 44]. In teleosts including the two medakas, however, no study reported FXYD3 and FXYD4 to date. Taken together, these results showed that the major expressed FXYD proteins of intestines were dissimilar between mammals and teleosts.

In the present study, the expression (mRNA and protein levels) of NKA α-subunit and FXYD12 were investigated in the Indian medaka and Japanese medaka. These results further confirmed that the *fxyd12* mRNA levels were the most abundant compared with those of the other *fxyd* genes in intestines of the two medakas. Meanwhile, the FXYD12 was co-localised with NKA in the intestinal epithelium of both medakas. No study has focused on the localization of teleostean intestinal FXYD12 to date, but intestinal NKA localization has been reported in teleosts. NKA exhibited in the basolateral membrane of intestines of both FW- and SW-acclimated teleosts [39, 45] because NKA has a crucial role to play in absorption of nutrient, ions, and/or water in all environments [31, 46]. In this study, similar NKA localization was observed between the two medakas. Taken together, FXYD12 and NKA were both expressed in the basolateral membrane of the intestinal epithelial cells of teleosts. In addition, by co-immunoprecipitation analysis, the results clearly exhibited that FXYD12 was associated with NKA α-subunit in the intestines of both medakas. The interactions between FXYD and NKA proteins, which is important for their modulatory mechanisms [14, 15] was also confirmed in the branchial and renal FXYD proteins of certain teleosts [18, 21–23, 25, 47]. Hence, this study showed predominant expression and interaction between NKA of FXYD12 in intestines of the two medakas, which implied that intestinal FXYD12 might play an important role in osmoregulation.

Upon salinity challenge, positive correlation of NKA expression (both mRNA and protein) was found in the intestines of Japanese medaka, showing significant increases in the SW-acclimated group. Recently, the six paralogs of NKA α-subunit were identified and investigated in the Japanese medaka [24, 48, 49]. Among them, *nka α1b* was the predominant isoform in the intestines [24, 48] and was significantly increased at 7 days after transfer of the Japanese medaka from FW to SW [48]. Meanwhile, no salinity effect was found on the mRNA expression of the other intestinal NKA α-isoforms [48, 49], except that *nka α2* exhibited temporary changes within 3 days post-transfer [49]. The present study assessed salinity effects on the protein abundance of total α-subunits. In the Japanese medaka, the increased protein abundance of NKA α-subunit found in the intestines might have resulted from enhanced mRNA abundance of *nka α1b*. In addition, similar patterns between protein abundance and activity of NKA were also observed in the intestines of Indian medaka. Being a closely related species to the Japanese medaka [27], the intestinal NKA expression of Indian medaka might be modulated via a similar pathway.

On the other hand, although salinity effects on FXYD12 expression were investigated in teleosts, these studies focused principally on the kidneys of limited species including these two medaka species [25]. In teleostean intestines, the expression patterns of *fxyd12* mRNA were only reported in the Atlantic salmon (*Salmo salar*) [20, 45] and spotted scat (*Scatophagus argus*) [16] upon salinity challenge. No significant difference in mRNA levels of intestinal *fxyd12* was found between FW- and SW-acclimated Atlantic salmon [20]. Similarly, in the spotted scat, intestinal *fxyd12* mRNA quickly decreased and subsequently recovered within 24 h after transfer from SW to FW [16]. However, in the Atlantic salmon and spotted scat, FXYD12 protein expression was not assessed and thus remains to be further elucidated [45]. This is the first study to investigate FXYD12 protein expression in teleostean intestines. The results of the present study showed that although intestinal *fxyd12* mRNA levels of both medakas increased with environmental salinities, the protein expression profiles of FXYD12 and NKA were parallel with those of NKA activity in respective medaka. Dissimilar expression patterns may have resulted from their osmoregulatory capabilities as described above. Moreover, our previous study reported that OdFXYD12 knockdown resulted in a decrease in NKA activity of medaka embryos, showing that the FXYD12 may play a role in enhancing NKA activity in medakas [25]. Taken together, these results revealed that the responses were different between the two medakas following salinity challenge and that the intestinal NKA activity might be directly affected by expression of mRNA and protein and/or by increasing via FXYD12.

Upon salinity challenge, expression of FXYD proteins and NKA has been investigated in the three osmoregulatory organs of the Indian medaka and Japanese medakas ([23, 25, 29], this study). Between these FXYD proteins in the two medakas, similar results were observed including tissue-specific distribution and parallel expression patterns with NKA. Tissue distribution analysis showed that FXYD11 and FXYD12 were expressed mainly and abundantly in the gills, kidneys, and intestines, respectively, of these two medakas. In each medaka, moreover, effects of salinity on expression patterns (including mRNA levels, protein abundance, and specific activity) between NKA and FXYD11 or FXYD12 were similar in the respective osmoregulatory organs. For the two medakas, these results not only emphasised again their similarity in phylogenetic relationship, but also implied that they might modulate NKA expression via the same FXYD protein in the respective osmoregulatory organs. These salinity-dependent expression patterns, especially NKA activity, however, were distinct between the two medaka species. These differences might be caused by their different osmoregulatory capabilities [27]. Although they are closely related species, they came from distinct natural habitats [27, 28]. The Indian medaka, residing in BW, showed higher NKA activities of gills and kidneys in the FW and/or SW groups [25, 29] as well as unchanged intestinal NKA activity among salinity groups. On the other hand, the Japanese medaka, being a FW inhabitant, revealed the highest NKA activities in the gills, kidneys, and intestines of SW individuals ([25, 29], this study). Therefore, the two medakas may use different tactics in their gills and kidneys, and similar strategies in their intestines to modulate NKA activity upon salinity challenge. In these processes for acclimation, FXYD proteins interacted with NKA, indicating they were involved in the osmoregulation via modulating NKA expression ([23, 25], this study). Previous studies reported that FXYD11 and FXYD12 of the two medakas might be able to enhance NKA activity [23, 25]. Hence, like the branchial FXYD11 and renal FXYD12, the intestinal FXYD12 may assist NKA to enhance its activity and result in providing a greater driving force to increase water absorption for maintenance of internal homeostasis.

## Conclusions

The present study investigated the FXYD12 expression in the intestines of the two closely related medakas which inhabit different salinity environments for illustrating the association between NKA and FXYD proteins of two medaka species in response to salinity changes. The data indicates that salinity effects on all protein expression patterns including protein abundance (both FXYD12 and NKA) and NKA activity were different between the intestines of the two medakas. Through protein interaction and co-localization of FXYD12 and NKA, the results clearly indicated that FXYD12 was associated with NKA α-subunit in the basolateral membrane of the intestinal epithelial cells of both medakas. Our results provide the potential modulatory mechanisms of intestinal FXYD12 for NKA expression in these two medakas in response to salinity changes and further clarify the molecular mechanisms of osmoregulation of euryhaline teleosts.

## Supporting information

S1 Table Probe construct used for quantitative real-time PCR. (PDF)

S1 Fig **Expression of *fxyd12* mRNA in various organs/tissues of the Indian medaka (Od; A) and the Japanese medaka (Ol; B).** Values are means ± SEM (N = 6 for the kidney and intestine and N = 1 for the others). G, gill; K, kidney; I, intestine; B, brain; E, eye; H, heart; L, liver; T, testis; O, ovary; M, muscle; CF, caudal fin; a.u., arbitrary units.
(TIF)

S2 Fig Alignment of putative amino acid sequences of the FXYD12 proteins in teleosts. The FXYD protein sequences belonged to different species, including Indian medaka (Od, *Oryzias dancena*), Japanese medaka (Ol, *O*. *latipes*), zebrafish (Dr, *Danio rerio*), spotted scat (Sa, *Scatophagus argus*), and Atlantic salmon (Ss, *Salmo salar*). The conserved residues are shown in Black background; the dotted line boxes indicate the specific epitope for antiserum. Accession numbers are: OdFXYD12, AGL34227; OlFXYD12, AGL39309; DrFXYD12, XP_002664620; SaFXYD12, AHB86582; SsFXYD12a, DAA06137; SsFXYD12b, DAA06138; SsFXYD12c, DAA06139.
(TIF)

S3 Fig Histology of intestines of the Indian medaka (Od) and Japanese medaka (Ol). The images of paraffin cross sections were obtained from the brackish water-acclimated Indian medaka (Od; A, B) and fresh water-acclimated Japanese medaka (Ol; C, D) with hematoxylin and eosin staining (HE; A, C) and periodic acid-Schiff staining (PAS; B, D). The PAS protocol used herein was modified from our previous study [25]. V, villus; *, lumen; Arrows, mucus cells. Scale bars: 20 μm.
(TIF)
