# Supplementary material for: Intestinal FXYD12 and sodium-potassium ATPase: A comparative study on two euryhaline medakas in response to salinity changes
Source: PLoS One. 2018 Jul 27;13(7):e0201252. doi: 10.1371/journal.pone.0201252 (PMC6063443; doi:10.1371/journal.pone.0201252)
Supplement: S1 Table — (PDF) [file pone.0201252.s001.pdf]

S1 Table. Probe construct used for quantitative real-time PCR.

Genes	Medaka	Primer sequence (5' to 3')		Accession numbers
fxyd5	Od	Forward	ACAGCCTGCGTGGATGAG	JX569227
		Reverse	TAAGCGTGGTGAGCAGGAAC	
	Ol	Forward	GTTGGATGAGGAGGAAGTGG	ENSORLT00000008380
		Reverse	TTGTGTTCCAGGTCGCATC	
fxyd6	Od	Forward	TCACTCCTGGTATGCGTGTC	JX624723
		Reverse	AGGCCAGTCCTCCAATTCTC	
	Ol	Forward	GTGTGCGTGTCAGCTGTTG	ENSORLG00000007198
		Reverse	AGAATTGGAGGACTGGCCTT	
fxyd7	Od	Forward	CTCCAAATCCAATTCCAACG	JX624724
		Reverse	CTGGTGCATAGTTGGTGGTG	
	Ol	Forward	AACCTTGCGGACAACAGG	JX565424
		Reverse	CGCGACAAACAAAACAACTG	
*fxyd*8	Od	Forward	CTCCTGCACTCGCTTCAG	JX569228
		Reverse	AGGAGGCAGAGTAGGACTGC	
	Ol	Forward	TCATTGTGTTGGTGGCATTC	JX565423
		Reverse	ACCGATTCGCAGAGATTCA	
fxyd9	Od	Forward	TGCAAGTTCAACCAGGACAA	JX569229
		Reverse	TTGGAGCTTAGCAGTTGCAG	
	Ol	Forward	GAAGATCTGCGCTTTGGTG	JX565422
		Reverse	ACCGCAGCAAGAATGAGG	
fxyd11	Od	Forward	GGCTCGTCATTGTCTGCTTG	JX624725
		Reverse	GGTCAGATCGCACTGCTAGA	
	Ol	Forward	CGGACTCTGTGTTGGTGAAG	JX624726
		Reverse	ATGACGAGGCCTCCAATTC	
fxyd12	Od	Forward	GGCGTTGTTGTGTTCTTGTC	JX569230
		Reverse	CTCAGTGCAGCTCAGTCATC	
	Ol	Forward	GACAGACGAGGCAGCATGT	JX643983
		Reverse	TCTGATCTTTGCTGGCATTG	
nka α	Od, Ol	Forward	GAACCGTCACCATCCTCTG	EU490421
		Reverse	GGCTGCCTCTTCATGATGTC	
β-actin	Od, Ol	Forward	CTGGACTTCGAGCAGGAGAT	EU490422
		Reverse	AGGAAGGAAGGCTGGAAGAG	
rpl7	Od, Ol	Forward	GTTCTGCAGCTTCTCCGTCT	NM_001104870
		Reverse	GAGCTCTCGCACAGACTTCA	

Od, Indian medaka; Ol, Japanese medaka; *nka α*, NKA α-subunit; *rpl7*, ribosomal protein L7.
