# Supplementary figures and images for: Intestinal FXYD12 and sodium-potassium ATPase: A comparative study on two euryhaline medakas in response to salinity changes
Source: PLoS One. 2018 Jul 27;13(7):e0201252. doi: 10.1371/journal.pone.0201252 (PMC6063443; doi:10.1371/journal.pone.0201252)

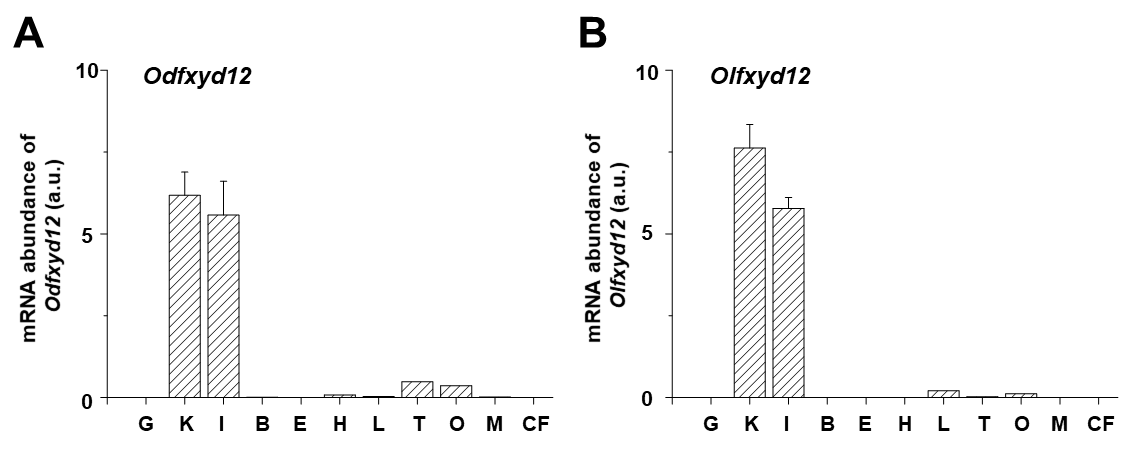

Supplement: S1 Fig — Expression of fxyd12 mRNA in various organs/tissues of the Indian medaka (Od; A) and the Japanese medaka (Ol; B). Values are means ± SEM (N = 6 for the kidney and intestine and N = 1 for the others). G, gill; K, kidney; I, intestine; B, brain; E, eye; H, heart; L, liver; T, testis; O, ovary; M, muscle; CF, caudal fin; a.u., arbitrary units. (TIF) [file pone.0201252.s002.TIF]

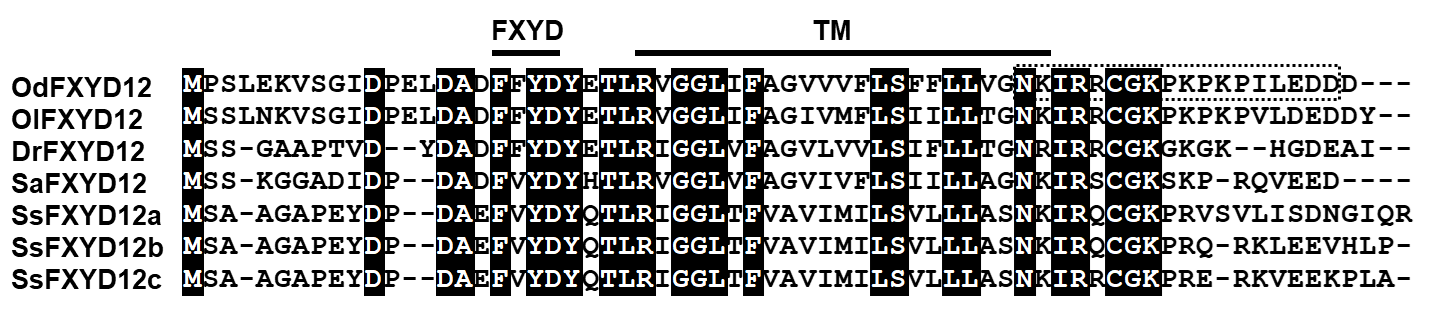

Supplement: S2 Fig — The FXYD protein sequences belonged to different species, including Indian medaka (Od, Oryzias dancena), Japanese medaka (Ol, O. latipes), zebrafish (Dr, Danio rerio), spotted scat (Sa, Scatophagus argus), and Atlantic salmon (Ss, Salmo salar). The conserved residues are shown in Black background; the dotted line boxes indicate the specific epitope for antiserum. Accession numbers are: OdFXYD12, AGL34227; OlFXYD12, AGL39309; DrFXYD12, XP_002664620; SaFXYD12, AHB86582; SsFXYD12a, DAA06137; SsFXYD12b, DAA06138; SsFXYD12c, DAA06139. (TIF) [file pone.0201252.s003.TIF]

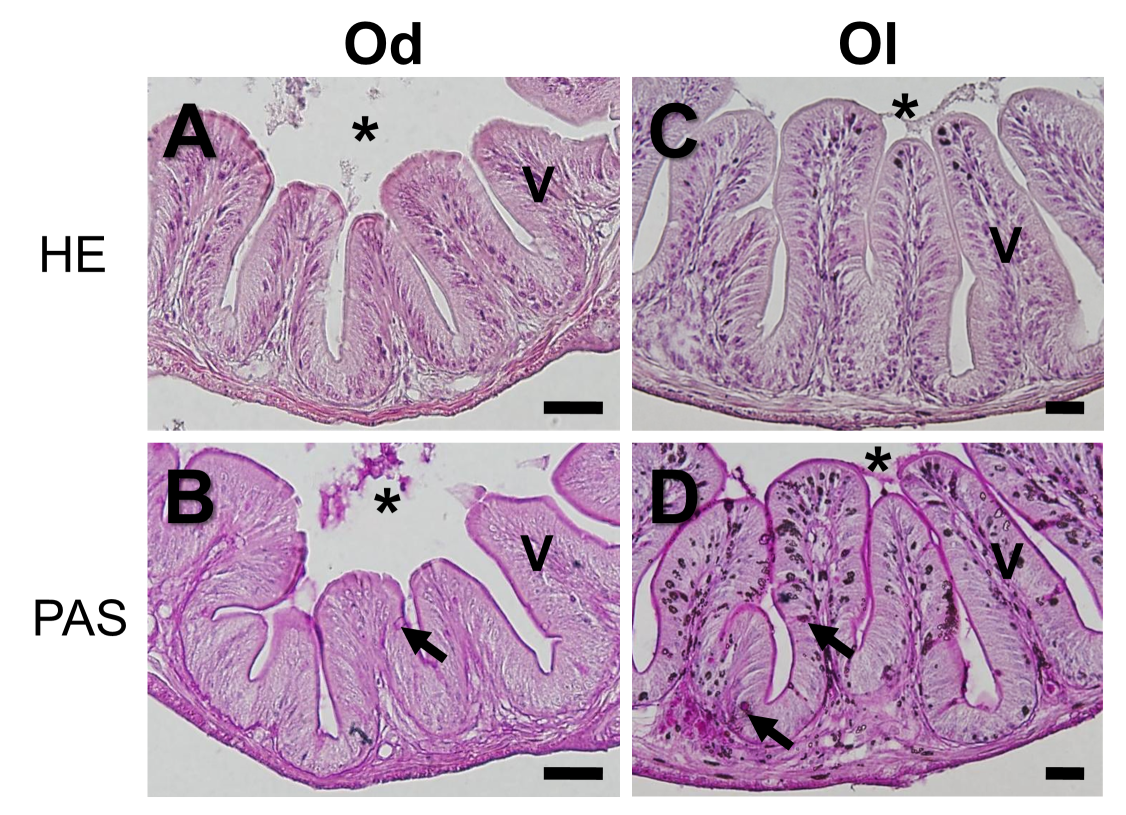

Supplement: S3 Fig — The images of paraffin cross sections were obtained from the brackish water-acclimated Indian medaka (Od; A, B) and fresh water-acclimated Japanese medaka (Ol; C, D) with hematoxylin and eosin staining (HE; A, C) and periodic acid-Schiff staining (PAS; B, D). The PAS protocol used herein was modified from our previous study [25]. V, villus; *, lumen; Arrows, mucus cells. Scale bars: 20 μm. (TIF) [file pone.0201252.s004.tif]
